# Resveratrol Action on Lipid Metabolism in Cancer

**DOI:** 10.3390/ijms20112704

**Published:** 2019-06-01

**Authors:** Terézia Kisková, Monika Kassayová

**Affiliations:** Department of Animal Physiology, Institute of Biology and Ecology, Faculty of Science, P. J. Šafárik University in Košice, Šrobárova 2, 041 54 Košice, Slovak Republic; monika.kassayova@upjs.sk

**Keywords:** resveratrol, cancer, lipid metabolism

## Abstract

Cancer diseases have the leading position in human mortality nowadays. The age of oncologic patients is still decreasing, and the entire scientific society is eager for new ways to fight against cancer. One of the most discussed issues is prevention by means of natural substances. Resveratrol is a naturally occurring plant polyphenol with proven antioxidant, anti-inflammatory, and anticancer effects. Tumor cells display specific changes in the metabolism of various lipids. Resveratrol alters lipid metabolism in cancer, thereby affecting storage of energy, cell signaling, proliferation, progression, and invasiveness of cancer cells. At the whole organism level, it contributes to the optimal metabolism extent with respect to the demands of the organism. Thus, resveratrol could be used as a preventive and anticancer agent. In this review, we focus on some of the plethora of lipid pathways and signal molecules which are affected by resveratrol during carcinogenesis.

## 1. Background

In the last decades, cancer diseases have reached the leading position in human mortality. The process of tumorigenesis includes changes at the cellular, tissue, and systemic levels. One of the main features of neoplastic transformation is altered metabolism. Rapidly proliferating cancer cells need more energy and consequently they metabolize more glucose to lactate than normal cells, in the presence of sufficient oxygen (Warburg effect) [1]. Malignant transformation is associated with an increased rate of intracellular glucose transport via glucose transporters (GLUTs) [2,3,4]. GLUT transporters use existing gradients in membrane sugar concentrations to translocate into the cell [5]. GLUT1 is slightly expressed in some human tissues, although high levels of it were found in many types of cancer [6,7,8,9,10]. The expression of GLUT1 is regulated by p53 via several mechanisms, such as reducing the expression of GLUT1 or inhibition of GLUT1 translocation to the plasma membrane, and, because of p53 deregulation during cancer, the levels of this transporter remain high [5]. GLUT4 is an insulin-sensitive transporter, expressed in some tissues such as muscle, heart, and adipose tissue. Elevated expression of GLUT4 is associated with many human tumors, e.g., head, neck, or breast tumors [11,12,13,14]. Finally, GLUT4, similarly to GLUT1, displays an interesting connection with cancer, as both transporters are transcriptionally repressed by p53 [15].

Fatty acid (FA) synthesis strongly depends on glucose through the generation of acetyl-CoA, a central metabolic precursor [16,17]. In tumor cells, most fatty FAs are synthesized de novo by fatty acid synthase (FASN) to arrange the intensive bioenergetics and structural changes. Indeed, FASN has been defined as a marker of cell proliferation and a drug target in oncology [18,19,20,21].

The occurrence of intense catabolism of sugar, lipid, and protein stores; body weight loss; and weakness leads to cancer cachexia syndrome [22,23,24,25]. Considering the intense lipid catabolism, high fasting triacylglycerols (TAGs) and low serum high density lipoprotein (HDL)-cholesterol were, for example, significantly associated with an increased breast cancer risk [26,27]. On the other hand, an increase of total cholesterol in the plasma could accelerate the development of tumors and is associated with the aggressiveness of the disease [28], together with increased low density lipoprotein (LDL) levels from patients with breast, colon, gastric, and ovarian cancer [27]; though, low total cholesterol levels have been considered as a risk marker for future cancer [29,30].

The altered tumor metabolism leads to the accumulation of specific metabolites in the tumor microenvironment and creates ideal conditions for tumor growth and metastasis. Low pH forms the tumor milieu and has positive effects on the migration of tumor cells [31,32,33]. Lactate also induces other factors important for tumor progression, such as CD44, hyaluronic acid, and transforming growth factor (TGF)-beta [34,35,36]. Furthermore, cytokines and growth factors released by fibroblasts and macrophages, some of the tumor infiltrating immune cells, could promote chronic inflammation and tumor progression [37,38,39,40]. On the other hand, cancer cells produce factors to attenuate immune cells, causing uncontrolled tumor growth [41,42]. A variety of metabolites is often deregulated within the tumors and supports the immune escape [43]. For example, the enhanced metabolism of L-arginine in myeloid cells declines the response of lymphocytes to tumor antigens, resulting in the failure of immune reactions and intensive tumor growth [44,45,46,47].

All of the bioenergetics and metabolic features not only permit cancer cells to survive under adverse conditions, but also allow their proliferation, progression, and invasiveness [3].

## 2. Resveratrol

Resveratrol (RES) is a well-known plant polyphenol nowadays. It has a stilbene structure and belongs to the group of phytoalexins that are produced under stress conditions in plants. According to its chemical structure, RES exists in two forms—cis and trans. Moreover, its isomers, adducts, derivates, and conjugates are intensively studied for their antioxidant and anti-carcinogenic effects. 

After an oral administration, RES accumulates in some organs, such as the kidney [48], intestine, or liver [48,49], which may probably relate to the places of its extensive absorption and metabolism. Our recent results indicate that RES also accumulates in breast tumors together with its main metabolites: 3-sulphate, and 4- and 3-glucuronide [50,51]. According to Bresciani et al., RES and its metabolites could accumulate in myocardial tissue [52]. However, no RES accumulation in tumor tissue of neuroblastoma in athymic mice was observed [53]. 

Due to its low water solubility, RES binds to proteins and protein transporters in the blood stream. It interacts with albumin, one of the plasma carriers, to catch the cell surface, or with lipoproteins in the order HDL < LDL < very low density lipoprotein (VLDL) [54,55]. RES absorption occurs by passive diffusion [56] or by a transport via ion channels [57] to pass the cell membrane, allowing its intracellular biological actions in the cell [58,59].

RES is a polyphenolic compound, playing its important role in many disorders. The extensive research of RES started through the “French paradox”, one of the most fascinating phenomena currently studied by scientists [60,61,62]. The French population has a relatively low incidence of coronary diseases despite its high intake of dietary cholesterol and saturated fat [63]. Moreover, oxidative damage and reactive oxygen species (ROS) action are involved in the pathogenesis of cardiovascular diseases [64]. The cardioprotective effects of polyphenols have been predicated to an increase in the plasma level of HDL cholesterol, protecting LDL from oxidation, a decrease in prostanoid synthesis from arachidonic acid, and the inhibition of platelet aggregation [65,66]. ROS formation causes oxidative damage to biomolecules, such as lipids, proteins, and DNA, resulting in many chronic diseases such as atherosclerosis, diabetes, cardiovascular diseases, and other degenerative diseases in humans [67]. Hydroxyl radicals damage cell membrane lipids via lipoperoxidation. Further, RES inhibits lipid peroxidation of LDL, prevents the cytotoxicity of oxidized LDL, and reduces platelet aggregation [68,69]. The antioxidant activity of RES may inhibit oxidation of LDL and, therefore, decrease endothelial damage associated with cardiovascular disease [70]. RES has been shown to act as an antioxidant by decreasing ROS generation in human [71,72,73] and animal models [74,75,76,77]. 

## 3. Resveratrol as a Modulator of Lipid Metabolism in Cancer

### 3.1. Resveratrol and Fatty Acid Synthesis

The role of FA metabolism, including both anabolic and catabolic reactions in cancer has gained increasing attention in recent years [78]. Lipid synthesis includes processes that convert carbons derived from nutrients into FAs. FAs are converted into diacylglycerides and TAGs via glycerol-3-phosphate to form the glycerol backbones of the lipids [79,80]. In healthy adults, de novo FA biosynthesis occurs in liver, adipose tissue, lactating breast tissue [81], or the brain [82]. However, in cancer cells, FAs are esterified to phospholipid (PL) for membrane lipid synthesis, promoting cell replication, rather than using TAG storage [83,84]. Indeed, it has been shown, that blocking of FASN in cancer cells results in cell growth arrest and induction of apoptosis [18,19,20,21]. RES significantly reduced lipid synthesis through the downregulation of FASN in many cancer cell lines [85,86,87]. Concomitantly, cell viability and mammosphere formation in breast cancer stem cells was significantly reduced (see Table 1) [87]. Growth arrest of pancreatic adenocarcinoma cells after RES application was associated with a significant decrease in glycogen breakdown and glucose carbon redistribution toward FAs by reducing FASN [83]. 

The expression of many genes involved in FA and cholesterol biosynthesis is activated via the phosphoinositide-3-kinase/protein kinase B/mammalian target of rapamycin (PI3K/AKT/mTOR) pathway [88,89,90]. It has been shown that RES could inactivate the PI3K/AKT/mTOR pathway and thus decrease the growth of various cancer cells in a dose-dependent manner [91,92,93]. For example, in glioblastoma-initiating cancer cells isolated from patients, RES in the doses of 5, 10 and 20 µM inhibited the invasion of these cells via downregulation of the PI3K/AKT/NF-κB signaling pathway in vitro and in vivo [85]. In HCT116 colon cancer cells, RES in the dose of 10–80 µM inactivated PI3K/AKT signaling via the upregulation of bone morphogenic protein, BMP7, and decreased the growth of these cells in a time- and dose-dependent manner [93]. In gastric MGC803 cells, RES caused a dose-dependent decrease in the protein levels of p-PI3K and p-PTEN (inactivate) and caused a cell cycle arrest in the G0/G1 phase [92]. In HeG2, Bel-7402, and SMMC-7721 hepatocellular carcinoma cells, RES inhibited the viability and proliferation of cancer cells and increased the apoptosis in a dose-dependent manner (20–200 µmol/L) via SIRT1 activation and concomitant inhibition of SIRT1-mediated post-translational modification of PI3K/AKT signaling [91]. Various agents inhibiting the PI3K/AKT/mTOR (PAM) pathway, such as rapamycin, are currently in various stages of clinical development in oncology, ranging from some in early phase evaluations to others that have already received regulatory approval for treatment in advanced cancers [94]. Rapamycin together with RES led to cell death in TSC^−/−^ MEFs bladder cancer cells, but not wild-type MEFs [95]. Combining rapamycin (20 nM) with RES (60 µM) had a synergistic effect in human multiple myeloma cells [96]. Moreover, PAM pathways play an important role in the synthesis and secretion of TAGs. However, RES as a potent inhibitor of the PAM pathway did not influence TAG concentration in the liver of female Sprague Dawley rats with breast cancer [97]. 

### 3.2. Resveratrol and Cholesterol Pathway

Another class of lipids, important for membrane function, is sterols, predominantly cholesterol and cholesteryl-esters. Cholesterol provides the structural backbone for the synthesis of steroid hormones, such as estrogen and progesterone [80]. 

A family of sterol regulatory element-binding proteins (SREBPs) is involved in FA and cholesterol biosynthesis [80]. Abnormally elevated cholesterol levels may be attributed to SREBPs mediated by 3-hydroxy-3-methyl glutaryl coenzyme A reductase (HMGCR) [98]. RES inhibited the mevalonate pathway, reduced HMGCR expression and activity, and decreased cholesterol synthesis in rat theca-interstitial cells [99]. Moreover, it has been found to inhibit lipid synthesis via SREBP1 inhibition in MiaPaCa-2 and Panc-1 pancreatic cancer cells in the dose of 50 µmol/L as well as in a transgenic mouse model of pancreatic cancer in the dose of 50 mg/kg body weight [100] or to reduce breast tumor volume concomitantly with the reduction of lipid content in serum in female nude mice in the dose of 22.4 mg/kg body weight [101]. 

SREBPs are also a target of the AMP-regulated protein kinase (AMPK) [102]. Professor Ido’s group revealed several mechanisms of RES action. First, it was the RES-induced activation of AMPK via SIRT1 activation [103]. They further supposed that the variability of this cascade may be responsible for the inconsistency of RES effects. They revealed that the effect of RES as a SIRT1 activator may not be solely via the activation of SIRT1, but also via an integrated effect of SIRT1-liver kinase B1 (LKB1)–AMPK. Thus, RES activates SIRT1 via direct binding to SIRT1 and through increasing nicotinamide adenine dinucleotide (NAD)^+^ levels by upregulating the salvage pathway through nicotinamide phosphoribosyl transferase (NAMPT) activation, an effect mediated by AMPK [104]. RES not only promotes deacetylation of a limited number of SIRT1 substrate proteins (for example, peroxisome proliferator-activated receptor gamma coactivator 1-alpha—PGC-1α) but activates other sirtuins in addition to SIRT1. For cancer treatment, RES may accelerate cell death if the cellular energy production is already impaired [104]. In ovarian A2780 and SKOV3 cancer cells and in female BALB/c nude mice with ovarian cancer, RES inhibited the growth of cells and tumors in vivo and induced apoptosis via increased expression and activation of AMPK and caspase 3, as well as decreased expression and activation of AMPK downstream kinase mTOR [105]. It has been shown that high doses of RES (4 g/kg body weight/day) activate AMPK in a SIRT1-independent manner, demonstrating that the dosage is a critical factor of RES functioning. Thus, RES-induced SIRT1 activation may play a role in the activation of AMPK both in vitro and in vivo [106].

Sirtuins are highly conserved NAD-dependent enzymes. The mammalian sirtuin family is involved in diverse cellular processes including DNA repair, lipid and glucose metabolism, and tumorigenesis [107]. The family consists of many sirtuin proteins, such as SIRT1, SIRT6, or SIRT7 localized predominantly in the nucleus; SIRT2 in the cytoplasm; or SIRT3, SIRT4, and SIRT5 localized in the mitochondria [108,109,110]. For example, SIRT1 deacetylates and destabilizes SREBP1, a hepatic transcription factor for lipogenesis and cholesterol synthesis [110,111,112,113]. SREBP2 controls cholesterol homeostasis via targeting the genes involved in cholesterol biosynthesis [114,115,116]. RES has been shown to modulate the expression and activity of sirtuins [117,118,119,120,121]. In gastric cancer cell lines, RES inhibited the viability and proliferation of BGC-823 and SGC-7901 cells in a SIRT1-dependent manner [122]. In MCF7 and MDA-MB-231 breast cancer cell lines, RES decreased breast cancer cell mass and viability in a dose-dependent manner, concomitantly with an increase in SIRT1 and SIRT3 protein content [123]. In colorectal cancer cells, RES stimulated the expression of SIRT1 in a dose-dependent manner, resulting in the downregulation of the nuclear localization of NF-κB and its related gene products, involved in tumor invasion and metastasis [124]. In hepatocellular cancer cell lines (HepG2, Bel-7402, SMMC-7721), RES activated SIRT1 protein and inhibited SIRT1-mediated post-translational modification of PI3K/AKT signaling [91]. SIRT2 activity mediated the inhibitory action of RES on the cell cycle of glioblastoma stem cells derived from human patients. Moreover, RES blocked the proliferation of glioblastoma stem cells without influencing the behavior of neural stem cells in a SIRT2-independent mechanism [125]. For more details, see Table 1. 

Lipid homeostasis may only be maintained if the excess of lipids that the cell has uptaken or synthesized can be metabolized or transported outside of the cell membrane [98]. Cholesterol-sensing liver-X-receptor (LXR) proteins are involved in maintaining cholesterol homeostasis. LXRs act to enhance the reverse transport of cholesterol from peripheral tissues via stimulating the expression of the ATP-binding cassette transporter A1 (ABCA1). These transport proteins direct cholesterol to apolipoprotein AI to form high-density lipoproteins. Apolipoproteins interact with lipids to form soluble lipid–protein complexes called lipoproteins. It is in this form that the major lipids—cholesterol, TAGs, and PLs—circulate in the plasma [126,127]. In addition to the known role of ApoA-1 as the key carrier of high density lipoprotein (HDL) and cholesterol receptor, it also enhances HDL influx and cholesterol efflux. Furthermore, it promotes excessive cholesterol excretion from peripheral liver tissue [98]. RES has been found to exert a biphasic effect on apolipoprotein M (apoM) in hepatoma cells and C57BL/6 mice. RES in the doses of 1 µM and 10 µM increased intra- and extracellular levels of apoM together with intracellular sphingosine 1-phosphatase (S1P) levels. However, at the higher dose of 100 µM it decreased extracellular apoM content [128]. But, nowadays there are not many publications dealing with RES action and apolipoproteins in cancer. RES action on apolipoproteins is rather studied with regard to obesity and atherosclerosis [129,130,131,132]. On the other hand, apolipoproteins are used as nanovehicles in targeted intracellular delivery of RES to glioblastoma [133] or breast cancer cells [134]. 

Low HDL and total cholesterol have been described as a marker of poor prognosis for current or future cancer [135,136,137,138]. Plasma levels of total cholesterol, LDL, VLDL, and TAG were significantly reduced in patients with benign breast disease in comparison with a healthy control group [139]. In breast cancer patients, the levels of total cholesterol and HDL were significantly lower, while VLDL and TAG levels increased markedly. Hence, higher levels of total cholesterol and HDL are associated with a reduction of breast cancer risk, whereas higher levels of VLDL and TAG are strongly associated with increased breast cancer risk [139]. RES (50 ppm) suppressed the serum TAG levels and VLDL and LDL cholesterol levels in hepatoma-bearing male Donryu rats, together with the suppression of hepatoma incidence and tumor growth. The data suggest that RES is a hypolipidemic agent with anticarcinogenic and antimetastatic properties [140]. 

### 3.3. Resveratrol, Ceramide, and Arachidonic Acid Pathway

Other lipids generated from FAs are sphingolipids, phosphoinositides, and eicosanoids [80]. Sphingolipids, including the two central bioactive lipids—ceramide and S1P, have opposing roles in regulation of cancer cell death and survival. While ceramide mediates cell death, S1P induces tumor cell proliferation, treatment resistance, and cancer metastasis [144,145,146]. Firstly, RES may act as an inhibitor of the S1P synthesis catalyzing enzyme—sphingosine kinase 1 and, thus, affect sphingosine kinase 1 expression and cell growth of breast MCF7 cancer cells [147]. On the other hand, RES has been shown to increase the intracellular concentration of ceramide, sphinganine, and sphingosine and the expression of enzymes related to the de novo ceramide synthesis pathway in hepatocellular HepG2cells. In addition, it reduced intracellular TAGs accumulation in lipid overload conditions [148]. In human gastric cancer cells, RES lead to cell cycle arrest and cell death through the sphingolipid metabolism pathway [149]. Moreover, 10 µM of RES for 24 h could modulate the lipidomic profile of Caco2 colon cancer cells (e.g., increase in diacylglycerol, TAG, phosphatidylcholine, phosphatidylinositol, and sphingomyelin species), leading to cell growth arrest [150].

Key enzymes in lipid metabolism are cyclooxygenases (COX-1 and COX-2), lipoxygenases (LOXs), and cytochrome P450 monooxygenases. COXs catalyze prostaglandin synthesis from arachidonic acid (ARA); in contrast LOX enzymes insert oxygen at the carbon of ARA [151,152,153]. ARA is polyunsaturated omega-6-fatty acid present in PL (phosphatidylcholine, phopshatidylinositides) of cell membranes and is generated for signaling purposes. The release of ARA from the cell membrane is initiated by phospholipase A2 (PLA2) and, thereafter, converted to eicosanoids [154]. Eicosanoids, including prostaglandins and leukotrienes, are products of local cell type-specific metabolism of ARA and form an important class of bioactive lipid mediators, playing critical roles in diverse physiological and pathological processes, such as inflammation and cancer [155,156,157,158]. In cancer patients, the level of PLA2 elevates significantly [159,160,161]. RES (5 mg/kg) inhibited the p38 MAPK-–cytosolic phospholipase A2–arachidonic acid–TxA2–[Ca^+2^]i cascade, resulting in the inhibition of phospholipase C and/or protein kinase C (PKC) activation and significant prolongation of platelet plug formation in mice [162]. In a plethora of other proteins and enzymes, RES inhibited cell viability via PLA2 decrease and sensitized breast cancer cells to doxorubicin therapy [163] or lead to p53-mediated cell death of prostate LNCAP cancer cells [164]. 

COX enzymes have been implicated in the development of malignant tumors. While COX-1 is expressed in vascular endothelial cells and contributes to angiogenesis, COX-2 is functional in tumorigenesis and tumor growth. The overexpression of COX-2 leads to cell escape from apoptosis and the cancer cells invade the matrix [165,166,167,168]. RES was able to block the expression and/or activity of COX-2 in many cancer studies in in vitro and in vivo conditions [51,169,170,171]. Zykova et al. found out that RES and its analogs directly bind with COX-2 and through this they inhibit COX-2 mediated prostaglandin production. Thereby, the colony-forming ability of human colon adenocarcinoma HT-29 cells was repressed [171]. In F344 rats, RES in concentrations of 1 and 2 mg/kg body weight, respectively, reduced NMBA-induced esophageal tumorigenesis by targeting COXs and thus influencing the levels of prostaglandin. In tumor tissue, the higher expression of COX-1, the upregulated COX-2 expression, and the increased levels of prostaglandin 2 were all significantly decreased by RES administration [172]. 

LOXs in mammalians, especially 12/15 LOX, modify cell membranes by peroxidation and on the other hand, 5-LOX produces signaling lipid mediators which exert effects via G protein-coupled plasma membrane-bound receptors. In some types of cancer cells, the LOXs are expressed constitutively and their activity is associated with cell proliferation, tumor angiogenesis, and metastatic potential [173,174]. RES (100 μg/rat) administered for 24 weeks significantly inhibited 5-LOX activity, reduced lipid peroxidation, and prevented DNA damage in DMBA-induced breast cancer in female Sprague Dawley rats [175]. RES was able to prevent apoptosis by inhibiting LOX and COX activity in the leukemia K562 cell line [176]. 

The superfamily of cytochrome P450 proteins is a large group of enzymes catalyzing many important biochemical processes, including steroid hormone, prostaglandin, and leukotriene biosynthesis. RES strongly inhibits the expression and activity of cytochrome P450 1A1 and 1B1 in many types of cancer cells [148,177,178,179,180]. Moreover, RES significantly attenuated the intracellular reactive oxygen species (ROS) formation and oxidative DNA damage as well as the cytotoxicity induced by the catechol estrogens [177].

As described above, eicosanoids are formed in reactions catalyzed by COXs, LOXs, and CYPs. This group of molecules encompasses a wide array of hormones which build the different classes, such as prostaglandins, thromboxanes, leukotrienes, lipoxins, epoxyeicosatrienoic acids, etc. Above all, the importance of prostanoid and leukotriene biosynthetic pathway in carcinogenesis and chronic inflammation is supported by a plenty of in vivo and clinical studies [156,157,181,182]. Prostaglandins and leukotrienes promote tumor growth by regulating tumor epithelial cells themselves and controlling the complex interactions between transformed epithelial cells and surrounding stromal cells to establish a tumor microenvironment that facilitates tumor-associated angiogenesis and evades attack by the immune system [182]. They may also lead to irreversible tissue damage [157]. In 1998, the chemopreventive potential of RES was examined by investigating its effect on eicosanoid production in mouse skin tumors [183]. RES significantly blocked PGE2 prostaglandin production, catalyzed by COX-1, proportionally to the RES concentration added to the reaction mixture. Moreover, the COX-2-induced production of prostaglandins PGE2, PGD2, and PGF2a was markedly decreased after RES treatment [183]. Sexton et al. revealed that concomitantly with the reduction of COX metabolites, PGE2 and PGF2α, the cellular levels of the phosphorylated/active form of antiapoptotic kinase AKT were decreased [184]. Further, the decreased expression and phosphorylation of AKT led to enhanced RES-induced cell death after mTOR inhibitor rapamycin in glioma cancer cells [185]. In pancreatic cancer, a relevant target is leukotriene A4 hydrolase. RES directly bound to this hydrolase and suppressed proliferation and anchorage-independent growth of pancreatic cancer by inhibiting leukotriene B4 production and expression of its receptor in a xenograft mouse model of human pancreatic cancer [186]. 

### 3.4. Resveratrol, Lipid Peroxidation, and Reactive Oxygen Species

Lipid peroxidation is a natural metabolic process under normal conditions and is one of the most investigated consequences of ROS actions on membrane structure and function. Lipid hydroperoxides and oxygenated products of lipid peroxidation as well as lipid peroxidation initiators (e.g., ROS) participate in signal transduction, cell proliferation control, and apoptosis [187,188,189]. RES prevents lipid peroxidation in many types of cancers [190,191,192,193,194]. On the other hand, damage to mitochondria induced by lipid peroxidation can lead to further ROS generation and in the presence of radicals, double bonds of FAs phospholipids can oxidize [195]. Treatment with RES leads to DNA damage and cancer cells death in vitro and in vivo in an ROS-dependent way [50,196,197,198]. Henceforward, healthy cells need more antioxidant activity; prooxidant mechanisms in cancer cells should inevitably lead to the cell death [199]. 

As mentioned previously, lipid mediators and other lipid molecules play a central role not only in cancer, but they are implicated in inflammation and tissue homeostasis. In addition to the cancer cells and their surrounding stroma the tumor microenvironment also contains innate immune cells such as macrophages, neutrophils, natural killer cells or dendritic cells, and adaptive immune cells (T and B lymphocytes). They communicate together by means of direct contact of cytokine and chemokine production and act in autocrine and paracrine manners to control tumor growth. The current available literature shows RES contradictory immunomodulatory effects in vitro and in vivo [200,201,202]. Though, Soto et al. confirmed its potential therapeutic activity with respect to tumor immunotherapy [203]. RES exerts its effects at multiple levels. These include lipoxygenases and cyclooxygenases synthesizing proinflammatory mediators from ARA, protein kinases such as PKC and PKD, lipid kinases, as well as I kappa B kinase α (IKK*α*), an activator of the NF-*κ*B pathway, which establishes a strong link between inflammation and tumorigenesis [204].

The key mechanisms in the tumorigenesis, such as the modulation of the metabolism, tumor growth, progression and metastasis to distant sites, the development of acquired treatment resistance, etc., take place in the tumor microenvironment [205,206,207]. This milieu consists of cancer cells and infiltrating immune cells, cancer-associated fibroblasts, angiogenic endothelial cells, or endothelial precursors, etc. [205,206,207,208]. The high rate of lactate causes pH lowering in the extracellular space [206]. Further, a hypoxic microenvironment inhibits β-oxidation of FAs in different tissues, resulting in the enhanced storage of TAG. Hypoxia-inducible factor 1 (HIF-1) promotes lipid accumulation, the uptake of free FAs and TAG production in liver and adipose tissue [81]. The up-regulation of HIF-1 results in tumor progression, followed by release of a variety of growth factors, cytokines and pro-angiogenic factors, such as vascular endothelial growth factor (VEGF) and GLUT-1 transporter [154]. RES shows an enhanced growth inhibitory and apoptotic potential at a pH lower than 7.5 in pancreatic cancer cell lines [209]. In hypoxic conditions at low pH, hypoxia-inducible factor HIF is activated. Its overexpression has been associated with the aggressiveness in many human tumors. RES in the dose of 100 mmol/L significantly inhibited HIF-1α protein accumulation concomitant with the reduction of VEGF-promoter activity and expression in human tongue squamous carcinoma cells [210]. However, VEGF expression after RES administration was not found to be eliminated in all in vivo experiments [211].

## 4. Clinical Trials

Clinical trials are predominantly focused on safety, adverse effects, and the overall tolerability of RES in human. There are several clinical trials describing the effects of RES in cancer patients, however, none of them describes some lipid mechanism involved in cancer. Nonetheless, the main problem of RES in human studies is its low bioavailability and possible side effects in the form of mild gastrointestinal discomfort, including diarrhea, nausea, flatulence, and abdominal discomfort [212]. Some review reports give an overview of clinical trials [213,214,215,216]. 

## 5. Conclusions

Preclinical and clinical evidence clearly shows that lipid metabolism plays a crucial role in tumor development and invasion. Numerous proteins which take part in lipid metabolism are also involved in cancer cell survival and proliferation. Many drugs and natural compounds are studied due to their ability to modulate lipid metabolism and thus influence the process of carcinogenesis. RES is a well-known natural substance showing some modulatory effects on lipid metabolism. RES alters lipid metabolism in cancer via various mechanisms and contributes to the optimal metabolism extent with respect to the demands of the organism. RES inhibits lipid synthesis via SREBPs inhibition, activates sirtuins concomitantly with the activation of AMPK, and downregulates the PI3K/AKT/mTOR pathway resulting in cancer cell apoptosis. RES decreases tumor volume and metastasis via decreasing serum TAG, VLDL, and LDL levels in cancer patients. Thus, it could be used in the modulation of cancer initiation and progression. However, further research is needed to use resveratrol widely in cancer management.

## Figures and Tables

**Table 1:** Main molecular mechanisms involved in resveratrol action regarding lipid metabolism in cancer.

Molecule	Cancer Type	Model	Dosage	Action	Ref.
FASN	Breast cancer	SKBR-3	5–150 µM (IC50 ~ 80 µM)	• decrease in FASN and Her2 expression in a dose-dependent manner	[86]
	Pancreatic cancer	MIA PaCa-2	50 and 100 µM	• cell growth arrest via significant decrease in glycogen breakdown and glucose carbon redistribution toward FAs by reducing FASN	[83]
SIRTUIN	Colorectal cancer	HCT116 SW480	1, 5, 10, 20, and 50 µM	• stimulation of the expression of SIRT1 in a dose-dependent manner • downregulation of nuclear localization of NF-κB, NF-κB phosphorylation and its acetylation, causing attenuation of NF-κB-regulated gene products involved in tumor invasion and metastasis	[124]
	Breast cancer	MCF7 MDA-MB-231	10, 25, and 50 µM	• decrease in breast cancer cell mass and viability in a dose-dependent manner • increase in SIRT1 and SIRT3 protein content	[123]
	Hepatocellular carcinoma	HepG2 Bel-7402 SMMC-7721	20–200 µmol/L	• inhibition of cell viability and proliferation and increase in apoptosis in a dose-dependent manner • activation of SIRT1 and inhibition of SIRT1-mediated post-translational modification of PI3K/AKT signaling	[91]
	Glioblastoma	GSCs derived from human biopsies	0–300 µM	• alteration of cell morphology after RES in the doses above 150 µM induction of GSCs necrosis • no effect on NSCs • blockade of SIRT2 activity or downregulation of SIRT2 expression with siRNAs counteracted the inhibitory effect of RES on cell proliferation	[125]
	Chondrosarcoma cancer	JJ012	5, 10, 25, 50, 100, and 200 µM	• increase in the protein expression of SIRT1 in a dose-dependent manner • significant reduction of the acetylation of NF-κB-p65 in a time-dependent manner (dose 50 μM)	[141]
		BALB/cA-nu (nu/nu) mice	50 or 100 mg/kg body weight	• reduction in size and weight of JJ012 tumors • reduction in tumor growth without affecting the body weight of the mice • increase in SIRT1 and cleaved caspase-3 expressions	[141]
SREBP	Prostate cancer	MiaPaCa-2 Panc-1	50 and 100 mmol/L	• inhibition of lipid synthesis via SREBP1 • enhancing the sensitivity of gemcitabine • reversed the gemcitabine-induced stemness	[100]
		LSL- Kras^G12D/+^ Trp53^fl/+^ Pdx1-Cre (KPC)	50 mg/kg body weight	• decrease in SREBP1 expression in tumor tissues • decrease in expression levels of the stem cell markers • decrease in PCNA protein synthesis	[100]
	Breast cancer	female nude mice	22.4 mg/kg body weight	• suppression of DCIS formation • reduction of tumor volume reduction in lipid content in serum • inhibition of SREBP1 and its downstream genes ACLY, ACC1, and FAS	[101]
		DCIS.com	50 and 100 µM	• suppression of CSCs growth • decrease in cell proliferation in CSCs in a dose-dependent manner • reduction of lipid content • inhibition of protein and mRNA level of SREBP1 and its downstream lipogenic genes • inhibition of mammosphere formation by CSCs	[101]
	Liver cancer	HepG2	15, 45, or 135 μmol/L	• reduction of intracellular lipid droplets • attenuation of hepatic steatosis • decrease of levels of intracellular TAGs enhancement of the phosphorylation of AMPK and downregulation of SREBP-1c and lipin 1	[142]
PI3K/AKT/mTOR	Colon cancer	HCT116	10–80 µM	• inactivation of PI3K/AKT signaling via upregulation of bone morphogenetic protein BMP7 • decrease of the growth of cancer cells in a dose- and time-dependent manner	[93]
	Breast cancer	SKBR-3	20, 40, and 60 µM	• inhibition of *AKT* phosphorylation • alteration of AKT/PI3K/mTOR pathway	[86]
	Hepatocellular carcinoma	HepG2 Bel-7402 SMMC-7721	20–200 µmol/L	• inhibition of cell viability and proliferation • increase in apoptosis in a dose-dependent manner • activation of SIRT1 and inhibition of SIRT1-mediated post-translational modification of PI3K/AKT signaling	[91]
	Gastric cancer	MGC803	6.25, 12.5, 25, 50, 100, 200, and 400 μM	• decrease in protein levels of p-PI3K and p-AKT in a dose-dependent manner • decrease in protein level of p-PTEN (inactive) in a dose-dependent manner • cell growth inhibition in a dose- and time-dependent manner • cell cycle arrested in G0/G1 phase	[92]
	Glioblastoma	U87 GSCs isolated from the patients BALB/c nude mice	0–100 µM 100 μg/mL	• deactivating oncogenic AKT and activating the tumor suppressor p53 gene network • inhibition of glioma cells and GSCs self-renewal and proliferation • reduction of tumor growth	[143]
		GSCs isolated from the patients	5, 10, and 20 µM	• inhibition of the invasion of GSCs via downregulation of the PI3K/AKT/NF-κB signaling pathway	[85]
		NOD/SCID mice	10 mg/kg body weight	• decrease in GSCs adhesion in a dose-dependent manner • suppression of GSCs adhesion in vivo	[85]

CSCs—cancer stem cells; DCIS—ductal carcinoma in situ; FASN—fatty acid synthase; GSCs—glioblastoma stem cells; NSCs—neuronal stem cells; SIRTUIN—silent mating type information regulation; SREBP—sterol regulatory element-binding protein; PCNA—proliferating cell nuclear antigen; PI3K/AKT/mTOR—phosphoinositide-3-kinase/protein kinase B/mammalian target of rapamycin.

## Abbreviations

ABCA	ATP binding cassette transporter A
AKT	protein kinase B
AMPK	regulated protein kinase
Apo	apolipoprotein
ARA	arachidonic acid
BMP7	bone morphogenic protein
COX	cyclooxygenase
FA	fatty acid
FASN	fatty acid synthase
GLUTs	glucose transporters
HDL	high density lipoprotein
HIF	hypoxia-inducible factor
HMGCR	3-hydroxy-3-methyl glutaryl coenzyme A reductase
IKKα	I kappa B kinase α
LDL	low density lipoprotein
LKB1	liver kinase B1
LOX	lipoxygenase
LXR	liver-X-receptor
mTOR	mammalian target of rapamycin
NAMPT	nicotinamide phosphoribosyl transferase
NF-κB	nuclear factor kappa-light-chain-enhancer of activated B cells
NAD	nicotinamide adenine dinucleotide
PAM	PI3K/AKT/mTOR pathway
PG	prostaglandin
PGC	peroxisome proliferator-activated receptor gamma coactivator 1-alpha
PI3K	phosphoinositide-3-kinase
PKC	protein kinase C
PL	phospholipid
PLA2	phospholipase A2
PTEN	phosphatase and tensin homolog
RES	resveratrol
ROS	reactive oxygen species
S1P	sphingosine 1-phosphatase
SIRT	silent mating type information regulation
SREBP	sterol regulatory element-binding protein
TAG	triacylglycerol
TGF-beta	transforming growth factor beta
VEGF	vascular endothelial growth factor
VLDL	very low density lipoprotein
